# Two-Step Separation of Nostotrebin 6 from Cultivated Soil Cyanobacterium (*Nostoc* sp.) by High Performance Countercurrent Chromatography

**DOI:** 10.3390/molecules19078773

**Published:** 2014-06-25

**Authors:** José Cheel, Petra Kučerová, Ian Garrard, Svetlana Ignatova, Pavel Hrouzek, Jiří Kopecký

**Affiliations:** 1 Department of Phototrophic Microorganisms-ALGATECH, Institute of Microbiology, Academy of Sciences of the Czech Republic, Opatovický mlýn, Třebon 379 81, Czech Republic; E-Mails: kucerova@alga.cz (P.K.); hrouzekp@gmail.com (P.H.); kopecky@alga.cz (J.K.); 2 Brunel Institute for Bioengineering, Brunel University, Kingston Lane, Uxbridge, Middlesex UB8 3PH, UK; E-Mails: Ian.Garrard@brunel.ac.uk (I.G.); svetlana.ignatova@brunel.ac.uk (S.I.)

**Keywords:** cyanobacteria, nostotrebin-6, countercurrent chromatography, polyphenolic compound

## Abstract

High performance countercurrent chromatography (HPCCC) was successfully applied for the separation of nostotrebin 6 from cultivated soil cyanobacteria in a two-step operation. A two-phase solvent system composed of *n*-hexane–ethyl acetate–methanol–water (4:5:4:5, v/v/v/v) was employed for the HPCCC separation. In the first-step operation, its neutral upper phase was used as stationary phase and its basic lower phase (1% NH_3_ in lower phase) was employed as mobile phase at a flow rate of 1 mL/min. In the second operation step, its neutral upper phase was used as stationary phase, whereas both its neutral lower phase and basic lower phase were employed as mobile phase with a linear gradient elution at a flow rate of 0.8 mL/min. The revolution speed and temperature of the separation column were 1,000 rpm and 30 °C, respectively. Using HPCCC followed by clean-up on Sephadex LH-20 gel, 4 mg of nostotrebin 6 with a purity of 99% as determined by HPLC/DAD-ESI-HRMS was obtained from 100 mg of crude extract. The chemical identity of the isolated compound was confirmed by comparing its spectroscopic data (UV, ESI-HRMS, ESI-HRMS^2^) with those of an authentic standard and data available in the literature.

## 1. Introduction

**Figure 1:**
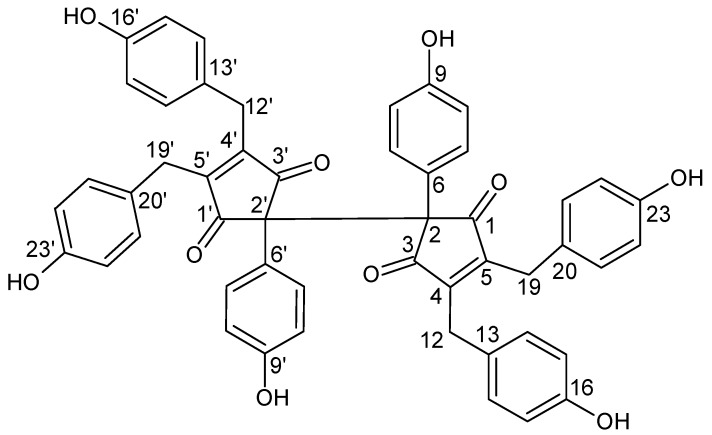
Chemical structure of nostotrebin 6.

Cyanobacteria (also known as blue-green algae) are Gram-negative microorganisms that represent the only group of prokaryotes that are able to perform oxygenic photosynthesis like plants. [1]. These micro-organisms are structurally diverse, and geographically widespread in freshwater, marine, and terrestrial habitats [2]. Recently, cyanobacteria have become an attractive source of innovative classes of pharmacologically active compounds showing a broad spectrum of biological activities ranging from antibacterial, antifungal, immunomodulatory, anticancer, antiviral and anti-inflammatory properties to UV-absorbing and protease-inhibiting effects [3]. The high degree of diversity in the bioactivities of cyanobacteria is due to the broad spectrum of its secondary metabolites [1,4,5]. Many of these are phenolic compounds that induce diverse biological effects in mammals [6]. Among these phenols, phenolic acids and their esters [7,8], a large group of phenols derived from phloroglucinol (phlorotannins) [9] and halogenated and sulphated derivatives of phenols [10] have been found (reviewed in [6,11]). Recently, nostotrebin 6 (NOS-6, Figure 1), a novel polyphenolic compound with a fully substituted 2,2'-bis(cyclopent-4-en-1,3-dione) skeleton, has been isolated from a methanolic extract of the cyanobacterial strain *Nostoc* sp. str. Lukešová 27/97 [12,13]. The structure of this compound was determined using X-ray crystallography and further supported by NMR, IR spectroscopy, and MS [13]. Nostotrebin 6 (NOS-6) has been reported as an effective inhibitor of acetylcholinesterase and butyrylcholinesterase under *in vitro* conditions [12,13]. Since the inhibition of these enzymes is currently used as a treatment strategy for Alzheimer’s disease (AD) [14,15], NOS-6 has relevant pharmacological potential for AD therapy. Therefore, development of a rapid and economical method for the preparative separation of NOS-6 is of great significance in order to speed up further pharmacological investigations and to provide amounts of NOS-6 that can serve as basic nucleus for the development of new semi-synthetic derivatives. So far, nostotrebin 6 has only been obtained from the biomass of cyanobacteium *Nostoc* sp. str. Lukešová 27/97 by using conventional separation methods, including precipitation and the use of polyamide and silica-gel columns, and HPLC [13]. Although these methods yielded a highly purified compound, but they implied time and solvent consuming operations, involved multiple steps and risk the loss of compounds due to the highly adsorptive effects of the solid matrices. High performance countercurrent chromatography (HPCCC) is a liquid-liquid partition chromatographic technique using a liquid stationary phase without solid support. Consequently, the method eliminates the complications resulting from the solid support matrix, such as irreversible adsorptive loss of sample onto the solid support, deactivation and contamination. The method produces high sample recoveries and permits the direct introduction of crude extracts into the column without additional sample preparation [16]. Countercurrent chromatography has been successfully applied to the separation of a number of natural products from higher plants [17,18,19,20,21,22] and cyanobacteria [23,24,25,26]. In this paper, we report an efficient HPCCC method for the separation of NOS-6 from soil cyanobacterium *Nostoc* sp. strain Lukešová 27/97.

## 2. Results and Discussion

### 2.1. HPLC/DAD-ESI-HRMS Analysis of the Crude Extract

**Figure 2:**
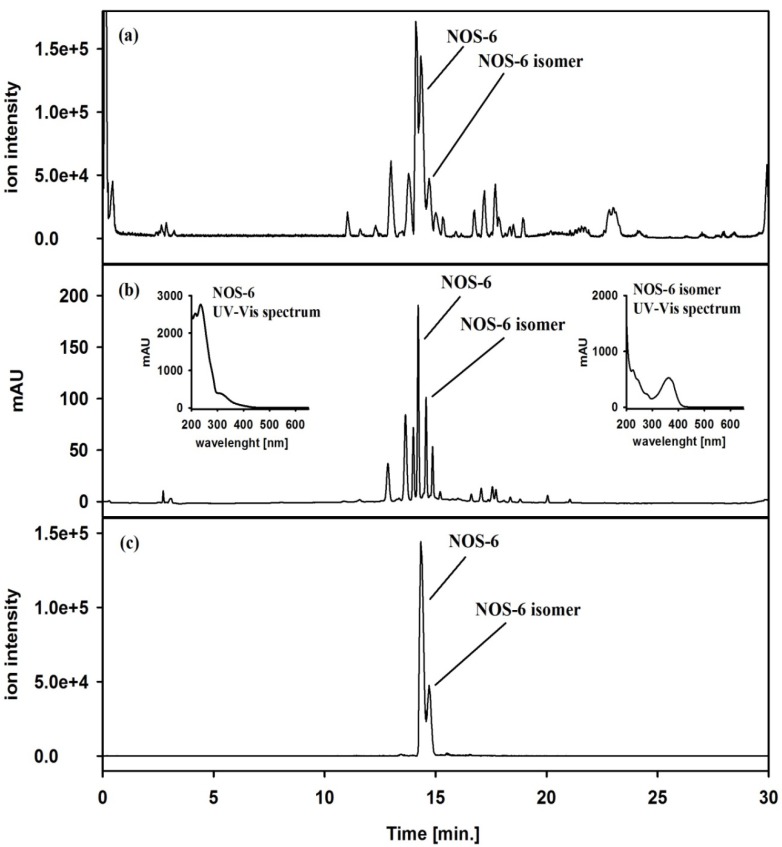
(**a**) Base peak chromatogram (BPC) of the crude extract from *Nostoc* sp. str. Lukešová 27/97 measured in the HPLC/ESI-HRMS operating in positive ion mode (mass range *m/z* 50–2000). (**b**) HPLC/DAD chromatogram (wavelength range from 200 to 650 nm) of the crude extract from *Nostoc* sp. str. Lukešová 27/97. (**c**) Extracted-ion chromatogram (EIC) of molecular ion at *m/z* 799 corresponding to NOS-6, operating in positive ion mode.

The crude extract obtained from the freeze-dried biomass of *Nostoc* sp. str. Lukešová 27/97 was first analyzed by HPLC/DAD-ESI-HRMS to confirm the presence of NOS-6. As shown in the base peak (BPC) (Figure 2a) and UV–Vis (Figure 2b) chromatograms measured in the HPLC system, the crude extract is highly complex, with predominance of hydrophobic constituents. The ESI-HRMS analysis provided information about the molecular weight of the extract components and the HRMS^2^ dissociations gave further structural information about the target compound. The peak at a retention time of 14.2 min (Figure 2a) showed a protonated [M+H]^+^ molecule at *m/z* 799 and its ESI-MS^2^ spectra revealed two fragment ions at *m/z* 307 and *m/z* 399, which were consistent with those of the MS spectral data of NOS-6 [13]. In addition, its UV spectrum (λ_max_: 214 and 235 nm) was in good agreement with those previously reported for NOS-6 [13]. The molecular ion of NOS-6 (*m/z* 799) was selectively monitored in the extracted-ion chromatogram (EIC) (Figure 2c). Collectively, these data confirmed the presence of NOS-6 in the crude extract. The occurrence of a minor peak at a retention time of 14.6 min (Figure 2a–c) with MS and MS^2^ spectra similar to those of NOS-6, but with a different UV spectrum (λ_max_: 225 and 362 nm, Figure 2b), suggests the presence of a NOS-6 isomer in the extract.

### 2.2. Optimization of HPCCC Conditions

A successful HPCCC separation largely depends on the selection of a suitable two-phase solvent system. Generally, these solvent systems need to satisfy the following requirements: (i) the settling time of the solvent system should ideally be shorter than 30 s to ensure the satisfactory retention of the stationary phase; (ii) the partition coefficient (*K*) of the target compounds should lie within the range 0.5 ≤ *K* ≤ 2.0 for efficient separation; and (iii) the separation factor (α) between two components (α = *K_2_*/*K_1_*_,_ *K_2_* > *K_1_*) should be greater than 1.5 [16,27]. The search for a suitable solvent system is the most difficult step because any change of the mobile phase composition is likely to change the stationary phase composition or volume; it is estimated that about 90% of the entire work on HPCCC has focused on this area [16]. A smaller *K* value elutes the solute closer to the solvent front with lower resolution while a larger *K* value tends to give better resolution, but broader peaks and more dilute peak fractions due to a longer elution time [16]. The higher the retention of the stationary phase, the better the peak resolution [28].

**Figure 3:**
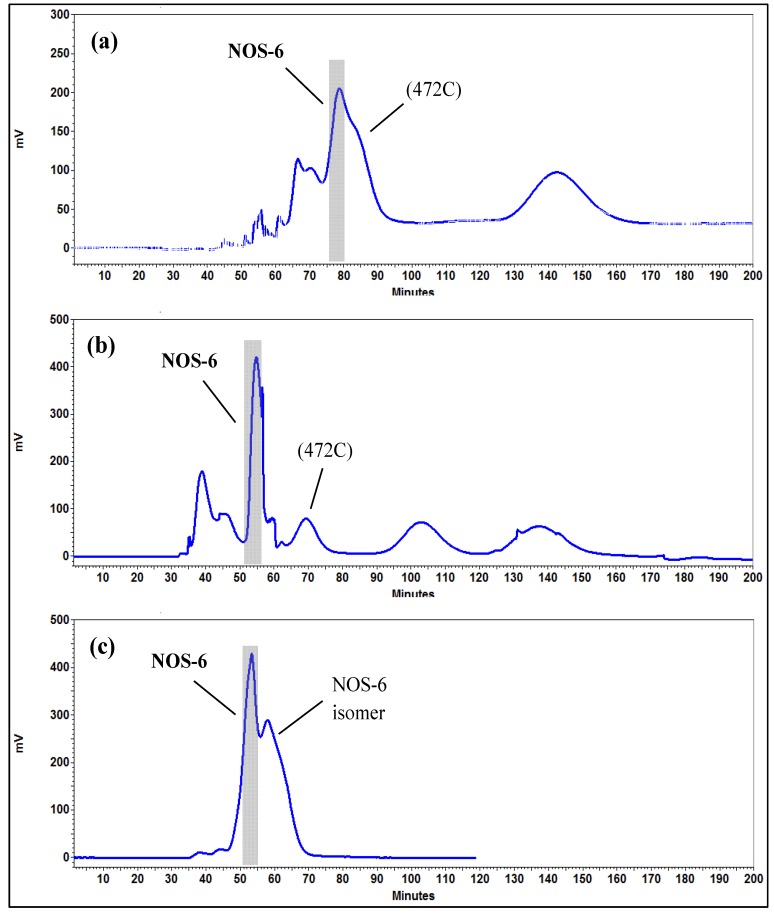
(**a**) HPCCC chromatogram of acetone-treated crude extract of *Nostoc* sp. Two phase solvent system: HEMWat, 4:5:4:5, v/v/v/v; mobile phase: lower phase; elution mode: reverse phase; flow rate: 1 mL/min; detection: 280 nm. (**b**) First-step HPCCC chromatogram of acetone-treated crude extract of *Nostoc* sp. Two phase solvent system: HEMWat, 4:5:4:5, v/v/v/v; stationary phase: neutral upper phase; mobile phase: base lower phase (1% NH_3_ in lower phase, pH 8.7); elution mode: reverse phase; flow rate: 1 mL/min; detection: 280 nm; 472C: contaminant (ion at *m/z* 472 from HPLC/ESI-HRMS analysis). (**c**) Second-step HPCCC chromatogram of target fraction. Two phase solvent system: HEMWat, 4:5:4:5, v/v/v/v; stationary phase: neutral upper phase; mobile phase: neutral lower and base lower phase (1% NH_3_ in lower phase, pH 8.7) as mobile phase with a linear gradient elution exchanged from 100:0 for 70 min and then to 0:100 for 130 min; elution mode: reverse phase; flow rate: 0.8 mL/min; detection, 280 nm.

Three different families of solvent systems composed of *n*-hexane-ethyl acetate-methanol-water (HEMWat), ethyl acetate-water, and ethyl acetate-*n*-butanol-water at different volume ratios [16,17,18,19,20,21,22,23,24,25,26,27,28,29] were examined to optimize the *K* values of NOS-6 (Table 1). Usually, there are several methods to determine the partition coefficient. For a crude sample mixture, HPLC/DAD can be used to measure the absorbance of an aliquot of each phase and compare the area under the peak between the corresponding peaks. However, some impurities can often co-elute with the target compound. In order to obtain an accurate partition coefficient of NOS-6, HPLC/ESI-HRMS was used for determining the *K* values. As shown in the Table 1, the system 3 (HEMWat, 4:5:4:5, v/v/v/v) provided a suitable *K* value (0.85, upper phase/lower phase) for the target compound and the shortest settling time (17 seg). However, when this solvent system was used for the HPCCC separation of NOS-6, the hydrodynamic equilibrium was disrupted at 25 min after having loaded the crude extract with the concomitant elution of emulsion-like substances. This drawback provoked the steady carryover of the stationary phase from the column, which significantly reduced the resolution and disturbed the tracing of the elution curves. This phenomenon has been reported to occur when working with extracts containing detergent-like compounds [16]. It is well known that cyanobacteria are rich sources of polar lipids (glycolipids and phospholipids) [30,31], which possess strong bio-surfactant properties [32]. Polar lipids could be removed from the crude extract by precipitation in cold acetone [33]. In the present study, when cold acetone-treated extract was subjected to HPCCC separation, the carryover of the stationary phase was practically reduced and the retention of the stationary phase was 76%. Nevertheless, the target compound peak eluted partly overlapped with a contaminant (Figure 3a), that was identified by HPLC/ESI-HRMS as a protonated molecular ion at *m/z* 472 (472C). Under these conditions, system 3 (HEMWat 4:5:4:5, v/v/v/v) afforded a suitable *K* value for the target compound, but it was poorly separated from 472C (*K* = 0.9) as these peaks had similar *K* values leading to an unsuitable separation factor (α = 1.06). Further experiments using different HEMWat solvent systems with similar polarity also failed to afford a satisfactory separation. Thus, improving the values of separation factors (α) between NOS-6 and 472C was necessary.

**Table 1:** The partition coefficient (*K*) of NOS-6 in different two-phase systems and the settling times.

System no.	Composition	Relative Proportions of Solvents (v/v/v/v)	Phase Volume Ratio	Settling Time (s)	Partition Coefficient (*K*)
1	*n*-hexane–ethyl acetate–MeOH–water	6:4:5:5	0.72	18	0.10
2	*n*-hexane–ethyl acetate–MeOH–water	5:5:5:5	0.67	19	0.21
3	*n-*hexane–ethyl acetate–MeOH–water	4:5:4:5	0.70	17	0.85
4	*n-*hexane–ethyl acetate–MeOH–water	3:5:3:5	0.82	23	1.47
5	*n-*hexane–ethyl acetate–MeOH–water	2:5:2:5	0.94	24	1.62
6	*n-*hexane–ethyl acetate–MeOH–water	1:5:1:5	1.00	23	3.53
7	EtOAc–water	5:5	0.86	20	12.40
8	EtOAc–*n-*BuOH–water	4:1:5	0.91	60	23.71
9	EtOAc–*n*-BuOH–water	3:2:5	0.99	38	16.57
10	EtOAc–*n*-BuOH–water	2:3:5	1.09	55	17.53

It is well established that for compounds with ionisable functionalities such as carboxylic and phenolic hydroxyl groups, changes in the pH values may significantly influence their chromatographic behavior. Indeed, the pH-related countercurrent chromatography has been successfully used for the separation of phenolic compounds [34,35]. With phenolic hydroxyl groups in the molecule, NOS-6 is a kind of weakly acidic compound, so its chromatographic behavior may vary by changing the pH value of the mobile phase in HPCCC. As presented in Table 2, a change in the *K* value of both NOS-6 (*K* = 0.51) and 472C (*K* = 0.80) was achieved by adding NH_3_ to the lower phase of the system 3 (1% NH_3_ in the lower phase, pH 8.7), which resulted in a satisfactory separation factor (α) between these constituents (α = 1.57). Thus, a good HPCCC separation of NOS-6 was achieved when the neutral upper phase of the HEMWat (4:5:4:5, v/v/v/v) was used as stationary phase while the base lower phase was used as mobile phase at a flow rate of 1 mL/min (Figure 3b). Interestingly, this modified mobile phase enhanced the retention of the stationary phase up to 84%, by increasing the difference in density between the two phases (Table 2). A decrease in the *K* value for the target compound resulted in a shorter separation time. To further improve the purity of NOS-6, a second-step HPCCC operation was applied to the target fraction obtained from first-step HPCCC separation. In this case, the neutral upper phase of system 3 was used as stationary phase, whereas both its neutral lower phase and base lower phase (1% NH_3_ in the lower phase, pH 8.7) were employed as mobile phase with a linear gradient elution exchanged from 100:0 for 70 min and then to 0:100 for 130 min at a flow rate of 0.8 mL/min. Accordingly, NOS-6 fraction was obtained by a two-step HPCCC method (Figure 3b,c). The NOS-6 fraction peak eluted close to a minor peak (Figure 3c) with MS and MS^2^ spectral data similar to those of NOS-6, but with a different UV spectrum (λ_max_: 225 and 362 nm).

Other factors such as the revolution speed of the separation column and the flow rate of the mobile phase were also investigated. The results indicated that reducing the flow-rate and increasing the revolution speed could improve the retention of the stationary phase leading to better resolution. So the flow rate for the first-step and second-step HPCCC operations was selected as 1 mL/min and 0.8 mL/min, respectively. The revolution speed was set at 1,000 rpm.

**Table 2:** The partition coefficient (*K*) and separation factor (α) values of NOS-6 and 472C.

System no.	Partition coefficient (*K*)		Separation factor (α)	Density difference (LP−UP g/mL)
	*K ^a^*	*K ^b^*		
3	0.85	0.90	1.06	0.09
3 *^c^*	0.51	0.80	1.57	0.12

*^a^* *K* value of NOS-6. *^b^* *K* value of 472C (contaminant: ion at *m/z* 472 from HPLC/ESI-HRMS). *^c^* System 3 with base lower phase (1% NH_3_ in lower phase). LP: lower phase. UP: upper phase

### 2.3. Separation of Nostotrebin-6 by HPCCC and Structural Identification

**Figure 4:**
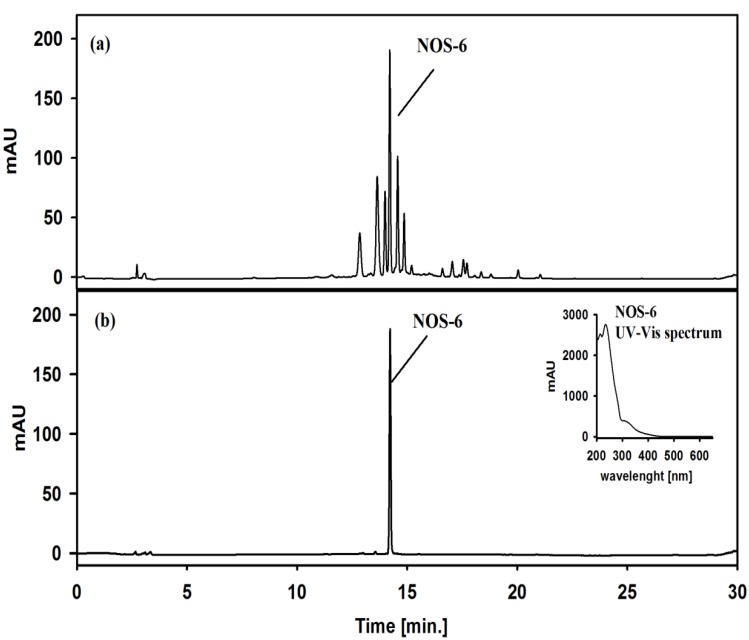
(**a**) HPLC/UV chromatogram (detection wavelength, 280 nm) of the crude extract from *Nostoc* sp. str. Lukešová 27/97 before separation by HPCCC. (**b**) HPLC/UV chromatogram (detection wavelength, 280 nm) of NOS-6 fraction obtained by two-step HPCCC separation.

**Figure 5:**
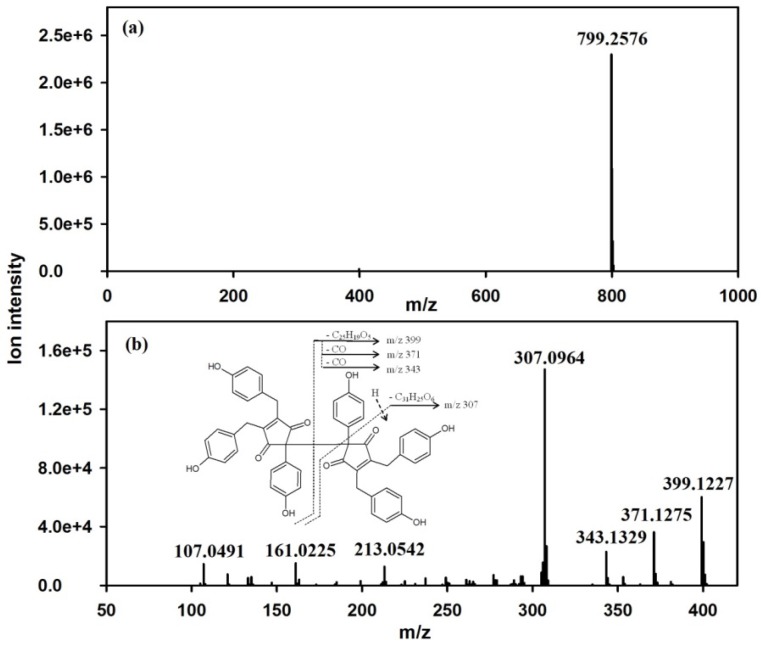
(**a**) Positive-ion mass spectra of NOS-6 fraction obtained by two-step HPCCC separation. (**b**) The MS^2^ spectra of the selected ion at *m/z* 799 corresponding to NOS-6.

Under the optimized conditions, 100 mg of crude extract previously treated with cold acetone was subjected to a two-step HPCCC method for separating NOS-6 in reverse phase mode with a run-time of 200 min. The stationary phase was retained at 84% relative to the total column capacity, after separation. The corresponding HPCCC chromatograms are shown in Figure 3b,c. The obtained HPCCC peak fractions were collected manually according to chromatographic peak profiles and then evaporated under reduced pressure for subsequent HPLC/DAD-ESI-HRMS analysis. From the first-step HPCCC operation (Figure 3b), seven peak fractions were collected. NOS-6 was identified in the peak fraction eluted at the retention time (R_t_) range from 51 to 56 min. From the second-step HPCCC operation (Figure 3c), two peak fractions were collected. The HPLC/DAD-ESI-HRMS analysis of the target fraction (R_t_ = 51–55 min) obtained from the second-step HPCCC showed a single peak that exhibited UV (λ_max_: 214 and 235 nm) (Figure 4b), MS (molecular ion at *m/z* 799 [M+H]^+^) (Figure 5a) and HRMS^2^ (fragment ions at *m/z* 399.1, 371.1, 343.1 and 307.0) (Figure 5b) spectral data, which accounted for NOS-6 [13]. The target fraction was further cleaned-up on Sephadex LH-20 gel. As a result, 4 mg of NOS-6 were obtained in two-step HPCCC separation at 99 % purity, as determined by HPLC analysis (Figure 4b).

## 3. Experimental

### 3.1. Reagents

All organic solvents used for HPCCC were of HPLC grade and purchased from Scharlab S.L. (Barcelona, Spain) and Analytika (Prague, Czech Republic). Ammonia was of reagent grade from Penta (Prague, Czech Republic). Organic solvents used for extraction and HPLC analyses were obtained from Analytika (Prague, Czech Republic). Solutions were prepared using reverse-osmosis deionized water (Ultrapur, Watrex, Prague, Czech Republic).

### 3.2. Cultivation of Nostoc sp. str. Lukešová 27/97

The *Noctoc* sp. strain Lukešová 27/97 used was obtained from the culture collection of soil algae and cyanobacteria of the Institute of Soil Biology of the Academy of Sciences of the Czech Republic. Cultivation was carried out in an 8.0 L photobioreactor containing Allen & Arnon medium, using a semi-batch system, at the constant temperature of 25 ± 0.5 °C, with continuous illumination (351 μmol m^−1^·s^−1^). The medium was stirred using a flow of mixed air and CO_2_ (98:2; V/V). After depletion of nitrates from the cultivation medium, the biomass from four-fifths of the total cultivation medium volume was harvested by centrifugation and then lyophilized. The remaining one-fifth of the suspension was refilled with freshly prepared Allen & Arnon medium. The cultivation was repeated several times in order to get enough freeze-dried biomass of *Nostoc* sp. str. Lukešová 27/97.

### 3.3. Preparation of the Crude Extract and Sample Solution

Freeze-dried biomass of *Nostoc* sp. str. Lukešová 27/97 (some 30 g) was homogenized with sea-sand and then extracted with methanol (500 mL). The extraction procedure was then further repeated with methanol (500 mL), three times. The resulting methanolic extracts were combined and evaporated to total dryness under reduced pressure at 40 °C yielding 4 g of dried extract. A representative amount of dried extract (100 mg) was dissolved in cold (−20 °C) acetone (50 mL) and stored overnight to precipitate polar lipids [33]. The suspension obtained was separated from the precipitate by centrifugation (5,000 rpm, 10 min), and the precipitation was twice repeated with cold acetone (50 mL). The supernatant was evaporated until dry and finally stored in the refrigerator for the subsequent HPCCC separation of NOS-6.

### 3.4. Apparatus

#### 3.4.1. Countercurrent Chromatography

The separation by high performance countercurrent chromatography (HPCCC) was performed on a semi-preparative apparatus of Model Spectrum (Dynamic Extractions Ltd., Slough, UK) consisting of two multilayer coil separation columns connected in series (PTFE bore tubing = 3.2 mm, total volume = 134 mL) and a 3 mL sample loop. The β-value range varied from 0.52 at internal to 0.86 at the external terminal (β = *r / R*, where r is the distance from the coil to the holder shaft and *R* is the revolution radius or the distance between the holder axis and central axis of the centrifuge). The revolution speed was adjusted with a controller to an optimum speed of 1,000 rpm. A Q-Grad pump (LabAlliance, State College, PA, USA) was used to fill the CCC apparatus with the stationary phase and elute the mobile phase. The effluent was continuously monitored by a Sapphire UV-VIS spectrometer (ECOM spol. s.r.o., Prague, Czech Republic) operating at 280 nm. The experimental temperature (30 °C) was adjusted by a H50/H150 Smart Water Chiller (LabTech Srl, Sorisole Bergamo, Italy). The EZChrom SI software platform (Agilent Technologies, Pleasanton, CA, USA) was used to record the HPCCC chromatograms.

#### 3.4.2. HPLC/DAD-ESI-HRMS

A Dionex UltiMate 3000 HPLC system (Thermo Scientific, Sunnyvale, CA, USA) equipped with a diode array detector (DAD) and high resolution mass spectrometry with electrospray ionization source (ESI-HRMS; Impact HD Mass Spectrometer, Bruker, Billerica, MA, USA) was used. The operating parameters of the mass spectrometer were as follows: the spray needle voltage was set at 3.5 kV, nitrogen was used both as nebulizing gas (2 bar) and drying gas (8 L/min), and the drying temperature was 200 °C. The scanning range was 50–2,000 *m/z* and the scanning rate 1 Hz operating in the positive ion mode. The DAD was set at 280 nm to record the peaks, and the UV–Vis spectra were recorded from 200 to 650 nm. A Phenomenex Kinetex C18 column (150 × 4.6 mm, 2.6 µm) was used for the analytical separation on the HPLC system.

### 3.5. Selection of the Two-Phase Solvent Systems for HPCCC

Three different solvent system families composed of *n*-hexane-ethyl acetate-methanol-water (HEMWat), ethyl acetate-water and ethyl acetate-*n*-BuOH-water were prepared using different solvent volume ratios (Table 1). The suitable two-phase solvent system was selected according to the partition coefficient (*K*) of the target compound and a proper settling time of the two-phase solvent system. HPLC/ESI-HRMS was used for determining the *K* values. By this way, the molecular ion of NOS-6 (*m/z* 799) was selectively monitored in the extracted-ion chromatogram (Figure 1c). The *K* values were determined by the following procedure: approximately 2 mg of crude extract was dissolved in 1 mL of each phase of the pre-equilibrated two-phase solvent system. After the test tube was shaken for 5 min, the solution was separated. The upper phase and lower phase were analyzed by HPLC/ESI-HRMS and the *K* values calculated according to the ratio of the peak areas from the selected ion. *K* = AU / AL, where AU was the peak area of selected ion in the upper phase, and AL, the peak area of the selected ion in the lower phase. The settling time of each solvent system was measured by a standard method [27]. Density difference between the upper and lower phases was also measured by weighing l mL of each phase with a micro balance.

### 3.6. Preparation of the Two-Phase Solvent System and Sample Solution for HPCCC Separation

The selected solvent system for HPCCC separation was prepared by adding all the solvents to a separatory funnel according to the proper volume ratios and thoroughly equilibrated by repeated vigorous shaking. After thoroughly equilibrated, the upper phase and lower phase were separated and degassed by sonication for 30 min shortly before use. The sample solution was prepared by dissolving the crude extract in 3 mL of the mixture of equal volume of lower phase and upper phase of the solvent system chosen for the HPCCC separation. The resulting sample solution was filtered through 0.45 μm membrane before use.

### 3.7. HPCCC Separation Procedure

The preparative separation of NOS-6 was performed by HPCCC in reverse phase mode using a two-phase solvent system composed of *n*-hexane–ethyl acetate–methanol–water (HEMWat system, 4:5:4:5, v/v/v/v). When operating the reverse phase elution strategy, the lower phase of the solvent system is pumped as the mobile phase and the upper phase as the stationary phase. The multilayer coiled column was initially filled with the upper phase (stationary phase). The apparatus was then rotated at 1,000 rpm, and the lower phase (mobile phase) was pumped into the column in reverse phase mode. After the mobile phase front emerged and hydrodynamic equilibrium was established, 3 mL of sample solution containing the crude extract was injected through the sample injection valve. The effluent from the outlet was continuously monitored at 280 nm. The temperature of the apparatus was set at 30 °C. In the first-step HPCCC operation the neutral upper phase of the system 3 (Table 1) was used as stationary phase and its base lower phase (1% NH_3_ in lower phase, pH 8.7) was employed as mobile phase at a flow rate of 1 mL/min. In the second-step HPCCC operation the neutral upper phase of the system 3 was used as stationary phase, whereas both its neutral lower phase and base lower phase were employed as mobile phase with a linear gradient elution exchanged from 100:0 for 70 min and then to 0:100 for 130 min at a flow rate of 0.8 mL/min. Peak fractions were collected manually according to chromatographic peak profiles and then evaporated under reduced pressure. The residuals were dissolved in methanol for subsequent HPLC/DAD-ESI-HRMS analysis. The separated compound was further cleaned-up by Sephadex LH-20 permeation with methanol as the elution system. The retention of the HPCCC stationary phase (*S*f) relative to the total column capacity was estimated from the volume of the stationary phase collected from the column after the separation was completed. The *S*f value was calculated using the following equation:

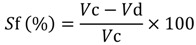
(1)
where *V*c is the known column volume and *V*d is the measured, displaced volume of stationary phase.

### 3.8. HPLC/DAD-ESI-HRMS Analysis and Identification of HPCCC Peak Fractions

The analysis of the crude extract and the HPCCC peak fractions was performed on a HPLC system equipped with DAD and ESI-HRMS detectors. The samples were subjected to a reversed phase column (Phenomenex Kinetex C18 column, 150 × 4.6 mm, 2.6 µm) at 30 °C. The mobile phase consisted of a combination of A (0.05% formic acid in acetonitrile, v/v) and B (0.05% formic acid in water, v/v). The gradient was as follows: 0–1 min, 85% B; 1–20 min, 85%–0% B; 20–25 min, 0% B; 25–30 min, 0%–70% B, at a flow rate of 0.6 mL/min. The DAD detector was set at a wavelength of 280 nm to monitor NOS-6. The obtained total ion chromatograms were evaluated for the presence of the protonated molecular ion [M+H]^+^ at *m/z* 799.5 corresponding to NOS-6. The chemical identity of the isolated compound was confirmed by comparing its spectroscopic data (UV, ESI-HRMS, ESI-HRMS^2^) with those of the authentic standard and data available in the literature.

### 3.9. Structural Identification

ESI-HRMS *m/z* 799.2576 [M+H]^+^ (calcd. for C_50_H_39_O_10_, 799.2543); ESI-HRMS^2^ [M+H]^+^ *m/z* (rel. int. %) 399.1 (42.5), 371.1 (20), 343.1 (15.0), 307.0 (100.0); UV λ_max_: 214 and 235 nm. The data obtained were consistent with those of the authentic compound and previously reported in the literature data for NOS-6 [13].

## 4. Conclusions

The overall results indicate that a two-step HPCCC method was developed and successfully applied for the separation of nostotrebin 6 from cultivated soil cyanobacteria. The separation method yielded a highly purified compound that was well suited for further pharmacological research, and also provided the reference for the scale up on a larger HPCCC column. The results demonstrated that HPCCC is a fast and efficient technique for the systematic isolation of bioactive compounds from cyanobacterial biomass.
